# Retrospective analysis of aeroallergen’s sensitization patterns in Edmonton, Canada

**DOI:** 10.1186/s13223-019-0320-y

**Published:** 2019-02-13

**Authors:** Hanan Ahmed, Maria B. Ospina, Kyriaki Sideri, Harissios Vliagoftis

**Affiliations:** 1 Department of Medicine, McMaster University, Hamilton, ON N2M 5E2 Canada; 2 Departments of Obstetrics & Gynecology and Medicine, Edmonton, AB T5H 3V9 Canada; 3 Division of Pulmonary Medicine, Department of Medicine, and Alberta Respiratory Center, University of Alberta, Edmonton, AB T6G 2R3 Canada

**Keywords:** Aeroallergens, Sensitization, Atopy, Skin prick testing

## Abstract

**Background:**

Sensitization to common environmental aeroallergens plays a significant role in the pathogenesis and severity of respiratory allergic disorders, specifically asthma and allergic rhinitis. Understanding sensitization patterns helps clinicians tailor care more effectively. This study examines patterns of sensitization to aeroallergens in subjects suspected of having an allergic disease in Edmonton and catchment area.

**Methods:**

Retrospective chart review of skin prick test (SPT) results to 11 environmental aeroallergens performed between January 1st and June 30th 2014 at a University-based clinic, where patients are referred for SPT by allergists, respirologists, otolaryngologists, internists and general practitioners. Potential differences in aeroallergen sensitization patterns were evaluated.

**Results:**

A total of 623 patients (36.9% males; 63.1% females), aged 4–84 years (mean age 38.6 years) had SPT done, of which 438 (70.3%) had a positive test for at least one aeroallergen (atopy). There were no significant sex differences in the frequency of atopy (males: 71.3% versus females: 69.7%; p = 0.373). The frequency of sensitivity to particular allergens among atopic subjects was: cat (53.1%), house dust mites (50.3%), grass (39.2%), birch (23.7%), alternaria (23.7%), dog (17.3%), poplar (12.1%), cedar (9.6%), aspergillus (9.6%), hormodendrum (8%), and penicillium (6.2%). Of 438 atopic patients, 110 (25.1%) were mono sensitized, 199 (45.4%) oligosensitized (2–3 allergens), and 129 (29.5%) polysensitized (≥ 4 allergens). There were no significant differences between males and females in the odds of being oligo-sensitized (OR: 0.95; 95% CI 0.58, 1.57). Polysensitization was significantly more frequent in males 37.2% than in females 24.8%; (OR: 0.95; 95% CI 0.58, 1.57).

**Conclusion:**

Cat is the most frequent perennial allergen and timothy grass pollen the most frequent seasonal allergen in Edmonton and catchment area. There was no significant difference in the frequency of atopy between males and females. However, males were more likely to be polysensitized compared to females.

## Background

Asthma and allergic rhinitis are common conditions, with significant morbidity and high economic burden on the individual and society [1]. It is estimated that 12% of children, and 8% of adults in Canada suffer from asthma, while 20–25% of Canadians have symptoms of allergic rhinitis [2]. The prevalence of allergic rhinitis continues to rise worldwide. The prevalence of asthma continues to increase in low- and middle-income countries as they develop into more industrialized countries, but is plateauing in developed countries [2, 3].

Complex interactions between genetic and environmental factors are central to the pathogenesis of respiratory allergic diseases. Studies have demonstrated that sensitization to aeroallergens is a risk factor for the development and severity of asthma [4, 5], especially in children [1, 4, 5].

Atopy is defined as IgE mediated sensitization to at least one environmental or food allergen and can be detected through skin prick testing (SPT). The numbers and types of aeroallergens an individual is sensitized to vary across geographic regions [6–8]. This could be explained by climate differences and the variability of allergen presence in various geographical areas. Studies have suggested that the type of sensitizing allergens may affect the course of allergic disease [9], which may also be affected by the number of allergens a patient is sensitized to. One study suggested that sensitization to one allergen (monosensitization) versus sensitization to multiple allergens (polysensitization) constitute two different phenotypes of allergic rhinitis [10]. Moreover, polysensitization seems to be associated with a more severe form of respiratory allergy [11]. Understanding sensitization patterns in certain geographic areas helps target specific interventions like allergen reduction and/or avoidance and encourages use of specific immunotherapy towards the most common allergens [12, 13].

The prevalence of atopy varies across cities and regions in Canada [14]. There are no studies to our knowledge, examining sensitization patterns in Edmonton; a region that has dry and cold long winters and short warm summers. This study examines the pattern of sensitization in subjects suspected of suffering from environmental allergies in Edmonton and catchment area. The catchment area is primarily Northern Alberta, but we also see a smaller number of individuals from Central Alberta, Northwest Territories, and Northern British Columbia.

## Methods

### Study design and study population

Retrospective chart review of SPT to environmental aeroallergens conducted at the University Lung Clinic in Edmonton (Alberta) between January 1st and June 30th 2014. The study was approved by the University of Alberta Health Research Ethics Board. The University Lung Clinic is an outpatient clinic affiliated with a tertiary care academic hospital. The clinic performs skin tests for patients suspected of allergy that are seen by allergists and respirologists within the clinic and also for patients suspected of allergy referred by other specialists (primarily respirologists, otolaryngologists, internists and family physicians) from the University Hospital and the community. The study population consisted of all patients of both sexes, that underwent skin testing for environmental aeroallergens during the study period. This represents a preselected population referred for SPT due to suspicion of allergy.

### SPT technique and definitions

A standardized panel of SPT was performed at the clinic for all patients suspected of sensitization to aeroallergens and included 11 common aeroallergens: timothy grass, birch, poplar, cedar, cat, dog, house dust mite (*Dermatophagoides pteronyssinus* and *Dermatophagoides farina*), alternaria, aspergillus, hormodendrum, and penicillium. SPTs to other allergens were performed when required by history and included, among others, horse, rabbit, cockroach, other pollens and food extracts, but were not included in the analysis because of the small number of patients tested with each allergen. Allergen extracts for skin tests were purchased from Omega Laboratories Ltd. (Montreal, Canada). Among them, only timothy grass, cat, *Dermatophagoides pteronyssinus* and *Dermatophagoides farina* extracts were standardized.

SPTs were performed by pulmonary function laboratory technicians under the supervision of the clinic allergists using a standardized technique. A drop of each allergen extract along with a drop from the positive (histamine) and negative (normal saline) controls was applied to the skin and then the skin was punctured through the drop using a standardized lancetters. The test was read 15 min later by the technician. A positive test was defined as an induration of at least 3 mm greater than the negative control within 15 min of application of the extract. Atopy was defined as having at least one positive skin test. Mono-sensitization was defined as sensitization to one aeroallergen of the panel of 11, oligo-sensitization as sensitization to 2–3, and poly-sensitization as sensitization to 4 or more aeroallergens.

### Data collection and analysis

Demographic information (age, sex) and SPT results were extracted from the medical records by one reviewer (HA) using a standardized data collection form. Data were analyzed using descriptive statistics using mean and standard deviations (SD) or median and interquartile ranges for continuous data, and proportions and percentages for categorical data. Crude odds ratios (ORs) with 95% confidence intervals (CI) were calculated to evaluate potential sex differences (reference group: males) in aeroallergen sensitization patterns. All data were analyzed using IBM SPSS Statistics for Macintosh, Version 23.0. Armonk, NY: IBM Corp.

## Results

A total of 627 patients underwent SPT to common environmental aeroallergens. Four patients were excluded from the analysis because of dermographism (1 case) or negative response to histamine (3 cases) leaving a total of 623 SPT results for analysis. The frequency of atopy among age and sex groups is illustrated in Table 1. There was no statistically significant difference in the frequency of atopy between males (71.3%) and females (69.7%) (OR: 0.92; 95% CI 0.64, 1.32). As shown in the table, individuals in the age group ≥ 66 years were the least likely to have positive SPT results (OR: 0.27, 95% CI 0.12, 0.61, p = 0.002, compared to the 19–35 group). All other groups had equal rates of atopy.

**Table 1:** The frequency of atopy among age and sex groups

Patient groups according to	n (% of total population)	% of atopy within the group
Sex		
Female	230 (37.7)	69.7
Male	393 (62.3)	71.3
Age (years)		
≤ 18	57 (9.1)	70.2
19–35	227 (36.4)	73.1
36–50	181 (29.2)	73.5
51–65	130 (20.8)	66.9
≥ 66	28 (4.5)	42.9

**Fig. 1:**
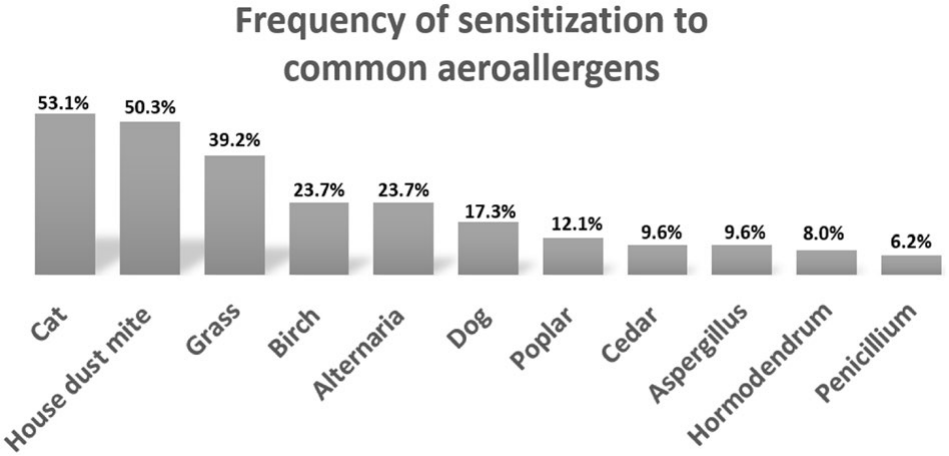
Frequency of sensitization to 11 aeroallergens among atopic individuals

Figure 1 represents the frequency of sensitization to all tested allergens. Cat was the most frequent allergen with 53.1% of atopic subjects testing positive, followed by house dust mite (50.3%). Timothy grass was the most frequent seasonal allergen (39.2%).

**Fig. 2:**
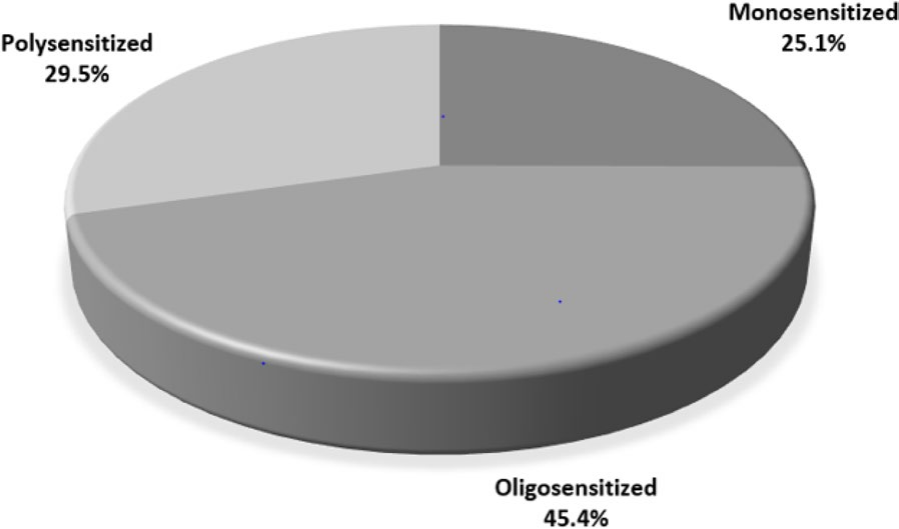
The frequency of mono-, oligo- and polysensitization among atopic individuals

Figure 2 presents the frequency of mono, oligo and polysensitization among the study population. Most patients were oligosensitized (45.4%) Among the patients who were monosensitized the top three allergens in frequency were house dust mite (14.7%), cat (11.6%), and timothy grass (11.0%). Sensitivity to cedar, hormodendrum and penicillium was never found in monosensitized individuals. 12 subjects were co-sensitized to hormodendrum and penicillium.

There were no significant differences between males and females in the odds of being oligo-sensitized (OR: 0.95; 95% CI 0.58, 1.57). Polysensitization was significantly more frequent in males (37.2%) compared to females (24.8%) (OR: 0.54; 95% CI 0.32, 0.91).

## Discussion

More than two-thirds of patients had skin reactivity to at least one aeroallergen. Our study population represents a selected group of patients referred for SPT likely due to a suspicion of allergy, which could explain the higher prevalence of atopy when compared to other Canadian cities (62.7%) [14], and the United States (54.3%) [15]. It is interesting that even in a highly selected population, 29.7% had no evidence of atopy. The absence of significant differences in the frequency of atopy between males and females and decreased likelihood of sensitization with advancing age were similar to the findings reported in other studies [14, 16].

In line with other studies, [11, 16] this study found that cat was the most frequent perennial allergen followed by house dust mite. We do not have information about cat ownership in our study population; however, there is evidence that 37% of households in Canada own one or more cats [17]. The exact figure of households with cats in Edmonton is not clear. The Edmonton Community Foundation reported that cats and dogs outnumbered kids in 2016 [18]. Airborne Fel d 1 (cat allergen) was detectable in undisturbed conditions in all homes with cats and in almost a third of homes without cats in a UK study [19]. The persistence of the cat allergen even after removal of the cat from the household for up to 20 weeks was confirmed in similar studies [20]. Patients may also be exposed to cat when visiting friends, family, or at workplace. The persistence of cat allergen may explain the high rate of sensitization to cat in our study population despite the lack of information about cat ownership.

House dust mite (HDM) survival depends on ambient humidity [21] and the main factor influencing the level of HDM allergens in homes is indoor humidity [22]. Active mites do not survive longer than 6–11 days at relative humidity ≤ 50%. HDM was reported among the top sensitizing allergens in various geographical regions with humid climates [8, 23–25]. To our knowledge, there are no studies describing HDM levels in Edmonton homes. Chan-Yeung et al. [22] reported that levels of dust mite allergens were low in 63 homes in Winnipeg, Manitoba, Canada, a city with a similar climate to Edmonton. House dust mite allergen levels in mattresses in Winnipeg were, depending on the season, between 25 and 50% of those found in mattresses in Vancouver, British Columbia, a city with much higher humidity. Seasonal variability in the levels of HDM allergen has been reported in a number of studies [22, 26]. The reason for the high, and in our view unexpected, prevalence of sensitization to HDM in our population is not clear. It is possible that patients acquired sensitization while living in other areas with higher environmental house dust mite levels, but the rates are quite high for the prevalence to be attributed primarily to this reason. It is also possible that the indoor environment in houses in Alberta in the winter has higher humidity, despite the general dry and cold winter atmosphere; houses are better insulated and have humidifiers associated with heating sources. Our study is limited by the lack of information on HDM levels in homes of sensitized individuals or even in Alberta homes in general, and a study addressing this issue would be important to assess risk of allergic sensitization to house dust mites in Alberta.

Grass pollen was the most frequent seasonal aeroallergen (39.2%) followed by birch pollen (23.7%) and alternaria (23.7%), a seasonal mold. Grass was reported among the top sensitizing allergens in other studies examining the frequency of aeroallergen’s sensitization in Italy [8, 25] and Quebec [16]. The grass season in Alberta runs from May to September [27], but during that time the levels can be high and patients are clearly symptomatic from exposure to grass pollen in the environment. Birch pollen counts are high in Alberta [27] and may play a significant role in respiratory allergy since 27.3% of our study subjects were sensitized.

The prevalence of sensitization to fungi, as determined by SPT, is estimated to be as high as 19% of the atopic population [28]. Alternaria was the most common mold detected by SPT in our study subjects (27.3% were sensitized to alternaria). In a study of subjects monosensitized to mold, alternaria was the most common (detected by SPT in 57.7% of subjects) [29]. Alternaria is a primarily outdoor mold with variable seasonal concentrations. Alternaria levels are detected in Edmonton from May to mid October [27].

Hormodendrum (Cladosporium), is primarily an outdoor mold that can also be detected indoors. Penicillium is an indoor allergen that can also be isolated from outdoor environments. Penicillium and hormodendrum are present in high counts in Edmonton from March to late Fall [27]. None of the subjects in our study were monosensitized to penicillium or hormodendrum. Large number of the subjects sensitized to fungi were sensitized to more than one of those included in our panel. This can be explained by the cross-reactive nature of fungal allergens, a finding that has been reported in other studies [29–31].

Similarly, there was no monosensitization to cedar (Cedrus), a genus of coniferous tress of the Cupressaceae family. Low levels of cedar pollen are detected April–July in Edmonton [27]. Because the counts are low, it is unlikely that cedar plays a significant role in allergy symptoms in that area. It is possible that low pollen counts make it a secondary rather that a primary sensitizer.

The MeDALL European birth cohort study suggested that monosensitization and polysensitization represent two distinct phenotypes with differences in symptoms and biomarkers [32]. Polysensitized individuals are more likely to have more severe and persistent respiratory allergic disease. Other studies confirm similar findings [10, 13, 33]. As has been reported in other studies, most patients in our cohort were oligosensitized [34, 35]. Based on the discussion above on the role of HDM in allergy in Alberta, it is interesting that HDM was the most common mono-sensitizing allergen in our study, as has been reported before [36]. The retrospective nature of our study and the lack of clinical information for the tested subjects do not allow us to make conclusions on the significance of this observation. Our results also showed males to be polysensitized more frequently compared to females. There are no studies to our knowledge about sex’ differences in the frequency of polysensitization and the physiological significance of this observation is not clear at this time.

## Conclusions

Our study shows that the three most common sensitizing aeroallergens in Edmonton and catchment area are cat, house dust mite and timothy grass. It is encouraging that immunotherapy for all three allergens has been shown to be effective [37–40]. The reason behind the high incidence of sensitization to house dust mites in our study is not understood, since house dust mite levels are expected to be low in Edmonton and surrounding areas. Further studies are needed to better understand the role of house dust mite in the development of allergic diseases in Alberta and similar environments.

There was no significant difference in the frequency of atopy between males and females, while rates of sensitization decreased with advancing age. Rates of mono and polysensitization did not seem to differ much from what has been shown in other studies, but males were more likely to be polysensitized compared to females. Further studies are needed to clarify the role of mono or polysensitization in the development and severity of respiratory allergic conditions.

## Abbreviations

SPT: skin prick test
HDM: house dust mite
